# Heterotopic Ossification in Breast Prosthesis

**Published:** 2015-01-23

**Authors:** Nuno Fradinho, Alice Varanda, João B. Martins, Pedro A. Martins

**Affiliations:** Department of Plastic and Reconstructive Surgery, Centro Hospitalar de Lisboa Central, Lisbon, Portugal

**Keywords:** heterotopic ossification, implant calcification, breast implant, breast surgery complication, implant contracture

## DESCRIPTION

An 80-year-old woman with a reconstructed breast 28 years ago presented prosthetic extrusion. Chest radiographs showed a round opacity with bone density. After implant removal, the breast maintained a rigid conformation with exuberant calcification. The massive calcification took the microscopic form of multilamellar crystal calcium deposits with true bone formation.

## QUESTIONS

1. **What is heterotopic calcification?**
2. **Is massive heterotopic ossification a frequent finding?**
3. **What are the patient risk factors for breast implant calcification?**
4. **What are the implant characteristics that are implicated with a higher risk of periprosthetic calcification?**

## DISCUSSION

In 1977, Peters et al described for the first time the calcification around breast implants and its mechanism of deposition. It is a common reaction that affects 16% to 25% of removed implants and it is associated with higher capsular contraction (Baker III and IV) and pain, although many cases remain subclinical. The implant capsule is formed by thick collagen fibers, inflammatory cells, histiocytes, and foreign-body giant cells; chondral metaplasia and hyalinization may coexist. It is thought that there is a time-related cascade of events until calcium deposits in the fibrous capsule: a strong histiocyte, macrophage, and foreign-body giant cell reaction leads to synovial metaplasia after a median time of 11.7 years and dystrophic calcification after 11 to 22 years.

Massive heterotopic calcification, characterized by an organized multilamellar deposition of hydroxyapatite crystals between collagen fibers, with osseous trabeculae surrounded by osteoblasts and lacunae osteocyte apposition, is a rare finding. There are few case reports describing this exuberant reaction in the literature.

The only host risk factor related to periprosthetic calcification is age more than 60 years at the time of surgery. The other factors identified are related to implant characteristics: the type of filling (saline or silicone), the implant generation, the duration of the implant in situ, and implant integrity. It appears to exist a causative relation between the rupture of the implant envelope (or its decomposition) with a higher deposition of calcium crystals, maybe by a more intense host reaction against silicone, Dacron, or polyurethane particles. Hundred percent of first-generation breast implants (1963-1972) led to calcification of the capsule after 14 years. Those implants had a thicker envelope and a Dacron patch; those characteristics were reviewed, and second-generation implants (1973-1987) had a thinner envelope and the Dacron patch was removed. That improved calcification rates: 0% before 11 years and 42% after 11 years in situ.

During the 1980s, the possibility of leakage of implants was of obvious concern, given the well-known problems with silicone gel breast injections made during the 1940s and 1950s. Several journals published articles about severe capsular contracture, heterotopic calcification, and serious connective tissue disorders after breast implant rupture or “bleeding” of silicone and silica particles. After the Food and Drug Administration's moratorium that banned silicone implants in the United States during the 1990s, manufacturers developed implants with a triple-layer shell that prevented leakage, and a more cohesive gel. These reintroduced implants (third generation) had almost no silicone leak, and a dramatic decrease in heterotopic ossification was noticed since then, making it a rare finding. Recent third-generation implants (after 1987) still lack large, randomized studies; however, the literature points to calcification deposits in 5% to 30% after 11 years in situ and 0% before that. Calcification takes 1 of 2 forms: globular aggregates and heterotopic bone. The former are found in saline prosthesis in the entire capsule surface and in silicone prosthesis restricted to the anterior surface; the latter was identified only in the anterior surface of silicone prosthesis. This patient presented an ulcer on a reconstructed breast with second-generation silicone prosthesis, 28 years before, following a radical mastectomy for breast cancer. The breast was very stiff, and the prosthesis could not be felt by external palpation. After prosthesis removal, the breast maintained the same rigid and nondepressible conformation. After careful dissection of the thin cutaneous flaps, a 1-mm-thick exuberant and dense calcification was identified under the capsule surface. The posterior wall of the prosthetic capsule was less involved. The mandatory capsulectomy was performed and the breast flaps were sutured to the anterior chest wall, under suction drainage.

## Figures and Tables

**Figure 1:**
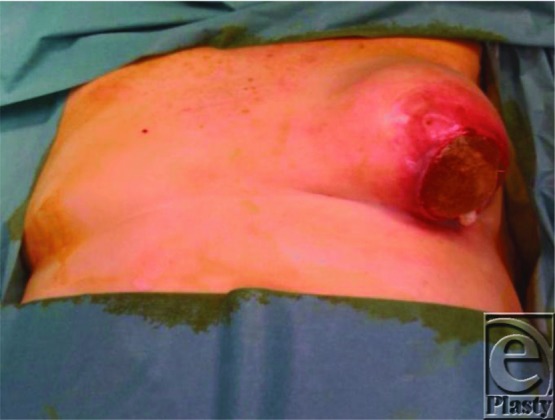
A 10-cm lower pole ulceration with left breast implant extrusion.

**Figure 2:**
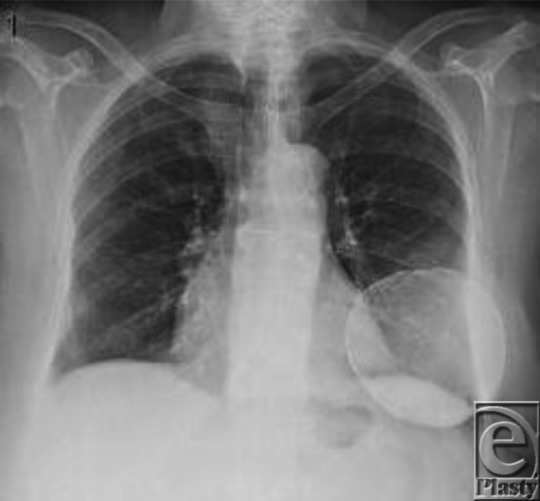
Chest posteroanterior identifies a bone density round calcification.

**Figure 3:**
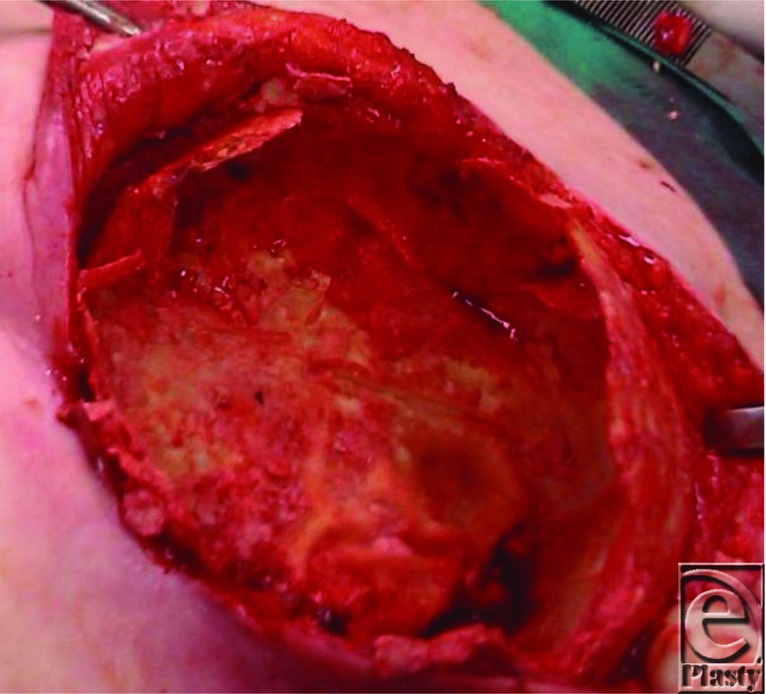
After careful dissection of the thin cutaneous flaps, a 1-mm-thick exuberant and dense calcification was identified under the capsule surface.

**Figure 4:**
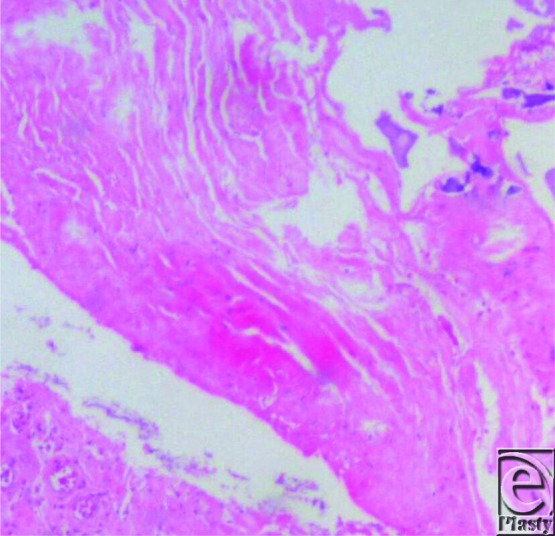
Hematoxylin and eosin coloration shows multilamellar crystal calcium deposits with true bone formation and osteocyte lacunae.
